# A cross-sectional survey and latent class analysis of the prevalence and clustering of health risk factors among people attending an Aboriginal Community Controlled Health Service

**DOI:** 10.1186/s12889-015-2015-8

**Published:** 2015-07-15

**Authors:** Natasha E Noble, Christine L Paul, Nicole Turner, Stephen V Blunden, Christopher Oldmeadow, Heidi E Turon

**Affiliations:** Priority Research Centre for Health Behaviour, School of Medicine and Public Health, University of Newcastle, Level 4 West HMRI Building, Callaghan, NSW 2308 Australia; School of Medicine and Public Health & Department of Rural Health, University of Newcastle, Callaghan, NSW 2308 Australia; Casino Aboriginal Medical Service, 43 Johnson Street, Casino, NSW 2470 Australia; Hunter Medical Research Institute and Faculty of Health, University of Newcastle, Callaghan, NSW 2308 Australia

**Keywords:** Clustering, Latent class analysis, Aboriginal Australians, Health risk behaviours, Multiple risk factors

## Abstract

**Background:**

Indigenous Australians are a socially disadvantaged group who experience significantly poorer health and a higher prevalence of modifiable health behaviours than other Australians. Little is known about the clustering of health risks among Indigenous Australians.

The aims of this study were to describe the clustering of key health risk factors, such as smoking, physical inactivity and alcohol consumption, and socio-demographics associated with clusters, among a predominantly Aboriginal sample.

**Methods:**

Participants (n = 377) attending an Aboriginal Community Controlled Health Service (ACCHS) in regional/rural New South Wales, Australia, in 2012–2013 completed a self-report touch screen health risk survey. Clusters were identified using latent class analysis.

**Results:**

Cluster 1 (‘low fruit/vegetable intake, lower risk’; 51 %) consisted of older men and women; Cluster 2 (‘risk taking’; 22 %) included younger unemployed males with a high prevalence of smoking, risky alcohol, and illicit drug use. Cluster 3 (‘inactive, overweight, depressed’; 28 %) was characterised by younger to mid aged women likely to have experienced emotional or physical violence.

**Conclusions:**

If future research identifies similar stable clusters of health behaviours for this population, intervention approaches targeting these clusters of risk factors should be developed and tested for Aboriginal and Torres Strait Islander Australians.

## Background

### Aboriginal and Torres Strait Islander health

Socially disadvantaged groups, which include many indigenous populations, experience poorer health and lower life expectancy compared to those less disadvantaged [24, 29]. The health of Aboriginal and Torres Strait Islander Australians, the Indigenous peoples of Australia, reflects this disparity [29]. Although the determinants are complex, one of the potentially modifiable contributors to the health gap is the disproportionately high prevalence of health risk factors including smoking, excess alcohol use, poor diet and physical inactivity [57]. Cancer screening [13, 42] and survival rates [5] are also known to be lower among Aboriginal and Torres Strait Islander than non-Indigenous Australians.

### Increased health risks associated with multiple risk factors

Health risk behaviours do not occur in isolation, and often co-occur or cluster together [15, 21, 40, 41]. Many health risk factors have a synergistic effect, where the combination of unhealthy behaviours increases the risk of disease or mortality more than the effects of the single risk factors [7, 40]. Thus there are likely to be subgroups within any population who are at higher risk of disease or death due to engaging in multiple health risk behaviours [40].

### Potential benefits of identifying clusters of risk factors

Information about whether, and which, risk factors group or cluster together can help inform preventive health efforts to avoid or reduce disease [21, 41]. Examining the clustering patterns of multiple modifiable health risk factors and demographics associated with health clusters allows targeting of health prevention interventions towards co-occurring risk factors and for the subgroups most likely to exhibit these risk clusters [22, 50].

### Clustering of health risks among Indigenous Australians

Data about the co-occurrence of risk behaviours among Aboriginal and Torres Strait Islander Australians are largely limited to identifying paired associations [3] or cumulative numbers of risk factors [8, 37, 53, 54]. Almost three-quarters (72 %) of Indigenous adults from non-remote areas reported two or more risk factors including smoking, risky alcohol consumption, physical inactivity and overweight/obesity [37]. Similar rates of multiple risk factors were reported for Indigenous Australians attending an urban Aboriginal Community Controlled Health Service (ACCHS) [53].

A more comprehensive understanding of the challenges facing Indigenous people, their communities and health services may be gained by identifying specific clusters of health behaviours. Exploring such patterns through techniques such as latent class analysis allows identification of individuals exhibiting common behaviours as well as characterisation of clusters by demographics.

There is a growing body of literature exploring risk clustering across countries and populations. For example, Verger reported five clusters among French adults, including a relatively healthy cluster and four unhealthy clusters characterised by poor diet, smoking, regular alcohol drinking, and binge alcohol drinking [55]. Among an Australian adult sample, French identified a ‘safe’ and a ‘moderate’ cluster, along with two risky clusters comprised of risky smokers and risky drinkers [22]. Only one previous study has looked at the clustering of health risk factors among Indigenous Australians [11]. Two clusters for Aboriginal adult men and women were reported: a ‘better’, and ‘worse’ cluster- characterised by hazardous alcohol intake, smoking and poor dietary choices [11]. However, a limited range of risk factors and demographics were included. Given the stark difference health gap between Indigenous and non-Indigenous Australians’, exploration of a wider range of health risks and socio-demographics is warranted.

The current study explored the clustering of a comprehensive range of risk factors and associated socio-demographics for a predominantly Aboriginal Australian sample. Depression was included as a ‘risk factor’ as it is a disease that affects many Aboriginal communities [9] and contributes both directly to the risk of cardiovascular disease (CVD) [38] and to lifestyle behaviours which increase the risk of developing CVD [51]. Differing results of health risk clustering studies across countries and in particular for different ethnic subgroups [7, 43] highlight the need to examine health risk clusters for different social and population groups [10]. Such information will help inform the planning and delivery of holistic preventive care efforts targeted towards co-occurring risk clusters and at-risk subgroups among people attending ACCHSs, with a focus on Aboriginal Australians.

### Aims

To examine among people attending an ACCHS:

i. The prevalence of self-reported health risk factors including high body mass index (BMI; overweight/obese), smoking, physical inactivity, risky alcohol consumption, inadequate fruit and vegetable intake, illicit drug use, depression, and under-screening for blood pressure, blood cholesterol, diabetes, and breast, cervical and bowel cancer;
ii. The clustering patterns of these health risk factors; and
iii. Socio-demographic characteristics (age, gender, Indigenous status, education level, employment status, and exposure to physical or emotional violence in the last 12 months) significantly associated with identified health risk clusters.

## Methods

### Study design and setting

An anonymous, cross-sectional health risk survey was administered on a touch screen laptop in two ACCHSs in regional and rural New South Wales (NSW). Sites were located in major towns, were staffed by Aboriginal and non-Aboriginal staff including doctors, nurses and allied health workers, and provided both on-site and outreach services to smaller or isolated communities. The two services had approximately 1250 and 2400 active patients (at least three visits in the last 2 years) respectively. ACCHSs provide culturally appropriate primary health care to Australian Aboriginal communities [17], with the majority of people who attend (74 - 86 %) being of Aboriginal or Torres Strait Islander origin [2, 4]. The two ACCHSs in this study represent the majority of those in NSW in terms of regional/rural location and patient numbers [4]. Ethics approval for the study was obtained from the University of Newcastle (Reference: H-2011-0153) and the Aboriginal Health and Medical Research Council of NSW (Reference: 806/11).

### Participants

Adults (≥18 years) attending the ACCHS for a general practice (GP) appointment who were physically and mentally able to provide informed consent and complete the survey were eligible. Aboriginal^1^ and non-Aboriginal people were invited to take part, on the assumption that non-Aboriginal people attending an ACCHS are likely have close ties to the Aboriginal community and are also therefore likely to share similar patterns of lifestyle and health risk behaviours.

### Procedure

Participants were approached by a Research Assistant (RA) in the waiting room and invited to complete the survey while waiting for their GP appointment. Assistance to complete the survey was offered as required. An Aboriginal RA assisted with patient recruitment for approximately half of the recruitment period, which occurred over four months in 2012 and 2013. Participants were asked to have their weight and height measured (optional), and were able to exit the survey if called in for their appointment. A RA recorded the estimated age and gender of non-consenting patients to assess consent bias. The survey was pilot tested with ACCHS staff and patients and refined prior to use. Items indicated that the touch screen survey was highly acceptable to people in this setting [34].

### Materials

The health risk survey was presented using Digivey Survey Suite software (CREOSO Digivey Survey Centre, Arizona, USA). The survey was designed using simple language and included pictures and limited text in order to improve accuracy and minimise reading demands. For example, a picture showing a standard drinks chart and the number of standard drinks in larger alcohol containers (*e.g.* cask of wine or carton of beer), was displayed to assist in answering questions about alcohol consumption. Survey software used branching algorithms to tailor questions to individual participants.

### Measures

#### Demographics

Age, gender, Indigenous status, highest level of education completed, employment status, and exposure to violence in the last 12 months were self-reported. Exposure to violence was assessed using two items: a) In the last 12 months, did anyone, including people you know, use physical force or violence against you? *(<yes>, <no>)*, from the NATSISS 2008; [1] and b) In the last 12 months, did anyone, including people you know, use emotional violence against you, *e.g.* insult you, swear or scream at you, or threaten to hurt you? *(<never>, <sometimes>, <often>)*, derived from the HITS screening tool [52]. Those who responded ‘yes’ to a) and/or ‘often’ to b) were classified as having been exposed to physical or emotional violence in the last 12 months.

#### Health risk factors

Key risk factors which contribute to the burden of disease and injury for Aboriginal Australians were included in the health risk survey [56]. The items used to assess health risk factors, and cut-offs used to dichotomise responses as ‘at risk’ or not at risk, are shown in Table 1.

**Table 1:** Description of items used to assess health risk factors, source of items and cut-offs used to classify participants as ‘at-risk’

Risk Factor and description of item used to assess risk	Cut-off used to classify ‘at-risk’ participants
**Body Mass Index (BMI)**	
Measured height and weight	BMI ≥ 25 kg/m^2^ (excluding pregnant women)
**Smoking status**	
Single item [12]; ‘Which of the following best describes your smoking?’	Current smokers (daily or occasional smokers)
**Risky alcohol use**	
Two items based on third question (AUDIT-3) of the AUDIT-C [6, 48] modified to current NHMRC guidelines [31]; ‘How often do you have more than 2/4 standard drinks in one day/ on one occasion?’	>2 stand. drinks daily or almost daily; and/or >4 stand.drinks weekly or more often
**Physical inactivity**	
Single item [46]; ‘Do you usually do at least half an hour of moderate or vigorous exercise on five or more days a week?’	<30 mins of exercise on five or more days per week [36]
**Fruit and Vegetable Consumption**	
Two items [12]; ‘How many serves of fruit/vegetables do you usually eat each day?’	< two serves of fruit; and/or < five serves of vegetables daily [33]
**Depression**	
Version of the Patient Health Questionnaire (PHQ-9) modified for use with Indigenous Australians [20]^a^	PHQ-9 score ≥ 10
**Illicit drug use**	
Participants were asked when they last used any illicit or illegal drugs	Any drug use in the last 12 months; including those who responded ‘prefer not to answer’
**Screening for blood pressure, cholesterol, diabetes and cancer: Underscreened**	
Participants were asked when they last had their blood pressure, blood cholesterol, and blood sugar (or HbA1c for those with diabetes) checked; and how long ago they had their most recent mammogram, pap test or bowel cancer test^b^	Not screened within recommended intervals for any age/gender appropriate screening test [30], including those who responded ‘can’t remember’

^a^We selected the modified version of the PHQ-9 as the tool’s authors suggested that the unmodified PHQ-9 [26] was unacceptable for use with Aboriginal and Torres Strait Islander people because of its wording and rating scale [20]. However, the authors also proposed including an additional item assessing anger (resulting in a total of 10 items), and in a subsequent validation study, using a cut-off score of 9, for the modified tool [19]. We did not include this additional anger item, nor use the modified cut-off score to classify possible cases of depression. This was due to a lack of psychometrics for the anger item and the small sample size of the scoring validation study (n = 34); ^b^Survey programming tailored these questions to the age and gender of participants and adjusted for more frequent screening requirements for those at increased risk. Those with a self-reported history of cervical, breast or colorectal cancer did not answer cancer screening questions

### Analysis

Participants with missing values were removed from analysis. A single ‘under-screened’ variable was created to dichotomise screening status: any participant who was not screened in accordance with guidelines for *any* of the relevant screening tests was classified as ‘under-screened’. For regression analysis, age was re-categorised into three broad groups: 18-34 yrs, 35-54 yrs and 55 + yrs. Analysis was conducted in 2013–2014.

Latent class analysis (LCA) was used to identify clusters of individuals with similar profiles of the eight health risk factors. LCA is a statistical tool used to identify homogeneous, mutually exclusive groups or classes within a heterogeneous population [32]. The latent class model aims to stratify observed variables by an unobserved or latent categorical variable that removes confounding between observed variables [27]. To account for uncertainty in class membership the model assigns each individual a probability of class membership. Each latent class is characterised by its estimated prevalence and the probability of individuals within that class exhibiting each of the health risk outcomes. The latent class regression model permits the inclusion of covariates to predict individuals’ latent class membership [27].

Goodness of fit and interpretability of the clusters were used to decide on the optimal number of classes. The LCA model was fit over a range of class numbers and the Bayesian and Akaike Information Criterion (BIC and AIC) generated for each (with lower BIC and AIC suggesting better goodness of fit) [16]. LCA analysis were performed in R 3.0.1 using the poLCA package [27, 39]. Stata (Statistical Software Release 13 College Station, TX: StataCorp LP) was used for data management and descriptive statistics.

## Results

### Sample

The consent rate was 69 %. There were no significant differences between the age and gender of consenters and non-consenters (*p*’s > .05; data not shown). Non-Aboriginal people were significantly more likely to consent, as compared to the proportions of active Aboriginal and non-Aboriginal patients registered as attending the ACCHSs, *χ*2 (1, N = 4091) = 9.71, p = 0.002. There were 377 surveys with complete demographic and risk factor data available for the LCA (27 surveys were excluded due to missing values; 3 of these due to refusal to have weight measured). Sample demographics are shown in Table 2.

**Table 2:** Demographic characteristics of study participants (n = 377)

Characteristics	n (% of sample)
**Gender**	
Male	149 (40 %)
Female	228 (60 %)
**Age**	
18-24 yrs	54 (14 %)
25-34 yrs	68 (18 %)
35-44 yrs	74 (20 %)
45-54 yrs	75 (20 %)
55-64 yrs	78 (21 %)
≥65 yrs	28 (7 %)
**Indigenous status**	
Aboriginal^a^	302 (80 %)
Non-Aboriginal	75 (20 %)
**Education**	
Year 10 or below (Primary, Year 9 or below, Year 10)	218 (58 %)
Year 12	56 (15 %)
TAFE^b^ course/Other	43 (11 %)
University or other tertiary	60 (16 %)
**Income source**	
Unemployed (Centrelink/Supported by family/Other)	248 (66 %)
Employed (FT/PT/Casual/Self Employed)^c^	129 (34 %)
**Exposure to Physical or Emotional Violence**	
No	78 (21 %)
Yes	299 (79 %)
**Data collection site**	
Site 1	178 (47 %)
Site 2	199 (53 %)

^a^Includes 7 participants who identified as either Torres Strait Islander or both Aboriginal and Torres Strait Islander;

^b^TAFE = Technical and Further Education, institutions which provide vocational education and training;

^c^FT = full time, PT = part time employment

### Prevalence of self-reported health risk behaviours

The risk factor profile of the sample is shown in Table 3. The most prevalent risk was inadequate fruit or vegetable intake (84 %), followed by being overweight or obese (69 %), inadequate physical activity, and being under-screened (each 52 %).

**Table 3:** Prevalence of self-reported health risk factors among the study sample

Risk factor	n (% of sample, [95 % CI])
Inadequate fruit/vegetable intake	316 (84 % [80, 88])
Overweight/obese^a^	260 (69 % [64, 74])
Inadequate physical activity	197 (52 % [47, 57])
Under-screened	194 (52 % [47, 57])
Current smoker	170 (43 % [38, 48])
Depression (using PHQ9 ≥ 10)	132 (35 % [30,40])
Risky alcohol intake	88 (23 % [19, 28])
Drug use in last 12 months^b^	78 (21 % [17, 25])

^a^BMI was measured and pregnant women were excluded. All other risk factors were self-reported;

^b^Includes those who responded ‘prefer not to answer’, n = 15

### LCA results: clustering of health risk factors

Models with one to four latent classes were estimated without regression variables. Based on the minimal AIC value, the 3-class model was the best fit for the data [AIC (3 class) = 3688; AIC (2 class =3697)]. The BIC indicated a 2-class model was the best fit [BIC (2 class) = 3753; BIC (3 class) = 3792]. The 2-class model consisted of two groups based on high prevalence of risky health behaviours, or high prevalence of overweight/obesity. The 3-class model presented a richer grouping of risk factors (discussed in detail below), including division of the overweight cluster into a sub-cluster distinguished by depression and lack of physical activity. The 3-class model was deemed to be more interpretable and was therefore decided to be the preferred model. When covariates were included in the latent class regression model, the 3 class model also had the minimal Chi-square goodness of fit value (*χ*^2^ = 274.80), indicating the preferred model on this criterion [27].

**Fig. 1:**
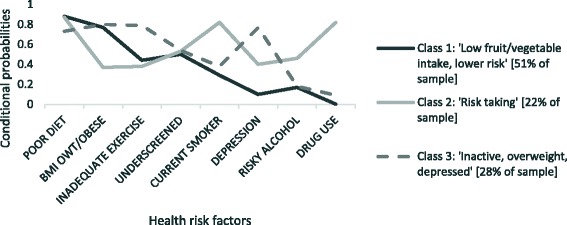
Conditional probabilities of each health risk factor associated with class membership

The conditional probabilities of each health risk outcome associated with the clusters are shown in Fig. 1. Clusters were named to best represent the health risk characteristics of each cluster. The three classes were characterised as follows:

*Class 1- Low fruit/vegetable intake, lower risk (51 %):* had the highest prevalence of poor diet characterised by inadequate fruit or vegetable intake, and a relatively high prevalence of being overweight, although not as high as class 3. Class 1 had the lowest prevalence of other risk factors including smoking, risky alcohol intake, drug use, depression, and under-screening.

*Class 2- Risk taking (22 %):* had the highest prevalence of behaviours including smoking, risky alcohol and drug use. Class 2 also had a relatively high prevalence of low fruit/vegetable intake, but was associated with the lowest prevalence of inadequate exercise and being overweight.

*Class 3- Inactive, overweight and depressed (28 %):* had the highest prevalence of inadequate physical activity, being overweight and depression. Class 3 had the lowest relative prevalence of having a poor diet, although the majority of this cluster still reported inadequate fruit and vegetable intake. Class 3 had a low to moderate prevalence of all the other risk factors including smoking, risky alcohol and drug use.

All three clusters were associated with a similar prevalence of under-screening.

### Regression results: predictors of class membership

Results of the latent class regression model are shown in Table 4, with class 1 (*low fruit/vegetable intake, lower risk*) as the reference group. Compared to class 1, classes 2 and 3 were characterised as follows:

**Table 4:** Socio-demographic variable odds ratios associated with membership of class 2 (*Risk taking*) and 3 (*Inactive, overweight and depressed*) relative to class 1 (*Poor fruit/vegetable intake, lower risk*)

	Class 2: *Risk taking*			Class 3: *Inactive, o/wt, depressed*		
Socio-demographics	Odds Ratio	Std. error	*p*-value	Odds Ratio	Std. error	*p*-value
**Gender** ^a^						
Male	3.11	0.46	0.01^*^	0.21	0.64	0.02^*^
**Age** ^b^						
35-54 yrs	1.40	0.46	0.47	3.35	0.66	0.07
55 yrs+	0.17	0.62	<0.01^*^	0.29	0.73	0.86
**Indigenous status** ^c^						
Non-Aboriginal	1	0.53	0.99	0.84	0.70	0.80
**Education level** ^d^						
Year 12	0.69	0.55	0.51	0.38	0.91	0.28
TAFE/Other	0.20	0.85	0.06	0.37	1.02	0.32
University/ Tertiary	2.37	0.64	0.89	2.61	0.60	0.11
**Employment** ^e^						
Unemployed	2.82	0.51	0.04^*^	2.48	0.54	0.09
**Exposure to violence** ^f^						
Yes	30.87	1.19	<0.01^*^	57.17	1.27	<0.01^*^
**Site** ^g^						
Site 2	0.92	0.40	0.83	0.81	0.47	0.66

^*^Significant predictors of class membership (*p* < .05)

^a^Gender reference group: Female

^b^Age reference group: 18-34 yrs

^c^Indigenous status reference group: Aboriginal participants (including seven participants who identified as both Aboriginal and Torres Strait Islander)

^d^Education level reference group: Year ten or below

^e^Employment reference group: Employed

^f^Exposure to violence reference group: No; Exposure to violence included having experienced any physical violence, and/or emotional violence often in the last 12 months

^g^Site reference group: Site 1

*Class 2-Risk taking:* had significantly higher odds of being male, being aged less than 55 yrs, being unemployed and of having been exposed to violence in the last 12 months. There was a non-significant trend for those in the risky cluster to have Year ten or below schooling compared to a TAFE/other qualification.

*Class 3-Inactive, overweight and depressed:* had significantly higher odds of being female and of having been exposed to physical or emotional violence in the last 12 months. There was a non-significant trend towards class 3 having higher odds of being aged 35-54 yrs compared to age 18-34 yrs.

## Discussion

Three distinct clusters of health risk factors, distinguished by socio-demographic characteristics, were identified within this predominantly Aboriginal sample. Aboriginal status was not a significant predictor of class membership, suggesting the risk patterns observed here may be a strong reflection of the overall social disadvantage of the client group of the ACCHSs, and that intervention approaches targeting the clusters reported here would be appropriate for the entire ACCHS client base (and not only for Aboriginal patients).

There is very little previous research exploring the clustering of risk factors among Aboriginal communities. Burke *et al.* identified a better and ‘worse’ cluster for both males and females [11]. Our study confirmed a clustering of alcohol, smoking and poor fruit/vegetable intake, but more strongly for males than for females. Additional clusters based on lower-risk plus poor fruit/vegetable intake, and physical inactivity, overweight, and depression, also emerged. As Burke *et al.* did not assess body mass or depression, their study could not have identified these additional clusters of risks factors.

Health risk cluster studies in other populations have tended to report a ‘healthy’ cluster, together with various numbers and types of unhealthy clusters [14, 16, 22, 25, 28, 43, 49]. Unhealthy clusters range from those based primarily on physical inactivity, [14, 25] poor nutrition, [16, 49, 55] risky alcohol, [22, 43, 49, 55] smoking, [22, 49, 55] to both smoking and risky alcohol [16]. Variable associations with physical inactivity have also been reported: Poortinga (2007) found that higher physically activity clustered with smoking and drinking, [40] while others have reported the opposite [49]. These disparate clustering results emphasise difficulties in comparing across studies using different analytical approaches, risk factors and definitions of risk [7, 40]. They further emphasise the potential lack of generalizability of such results, and the importance of conducting research for specific populations, such as Indigenous Australians.

Reported predictors of health risk clusters also vary across studies. Young males tend to be in clusters characterised by smoking and/or risky alcohol, [7, 10, 14, 49, 50, 55] or to have a greater numbers of risk factors [21] Women tend to be in healthier clusters, although some studies report female gender associated with clusters characterised by physical inactivity, [7] poor diet, [55] smoking, [22] or even with a more pronounced clustering of risky behaviours than men [40]. Older age tends to be associated with less risky behaviours, as does higher education, income, and other measures of higher socio-economic status [7, 10, 14, 16, 21, 22, 25, 28, 41, 44, 45, 49, 50].

Our demographic results are broadly consistent with these previous findings regarding gender and socioeconomic status, although there was no significant relationship between education level and cluster membership. Regarding depression and preventive screening, previous work reports an association between lower psychological distress or depression [14, 21, 45] and healthier clusters [55]. Lower compliance with preventive screening or medical check-ups was associated with clustering of other risky behaviours, [23] or with an ‘inactive’ cluster among a sample of women [25]. In contrast, we found that depression was associated specifically with inactive and overweight women, rather than with more risky behaviours or with poor diet. We found no substantial differences in screening behaviours across clusters [23, 25]. However, our measure of under-screening may not have been sensitive enough to reveal differences between clusters.

### Limitations

Several study limitations should be noted. Reliance on self-reported risk factor status may affect the accuracy of our results, including potential social desirability bias. Although validated measures were used where possible, [19, 46] many show only moderate sensitivity and specificity (such as short measures of physical activity or diet), and most have not been specifically validated for use with Indigenous Australians. The cut-offs used to dichotomise risk status (based on national guidelines) classified a large proportion of the sample as at-risk. Different clustering patterns may have resulted if we had restricted ‘at-risk’ status to, for example, obese participants, or to the consumption of less than five (vs seven) serves of fruit and vegetables per day. Finally, the small sample size, rural/regional setting, and inclusion of non-Aboriginal participants, may limit the generalisability of our results to other settings, such as for Aboriginal Australians living in urban areas.

### Implications for practice

Our clustering results support the idea that this population could benefit from interventions targeting multiple, related health risk behaviours, either simultaneously or sequentially [43, 49]. Alternatively, interventions aimed at addressing single risk factors may need to consider other risk factors which are likely to be present [22]. The relatively small variation in fruit and vegetable intake and under-screening across the sample suggests that almost all people attending an ACCHS would benefit from improved diet and screening. About a quarter of clients (typically younger, unemployed males), appear most likely to benefit from targeting risky behaviours such as smoking, alcohol and drug use; but with an aim to maintain healthy BMI and exercise levels. About a third of clients, particularly overweight women aged 35-55 yrs, may benefit from an intervention approach which targets, or at least considers, the impact of depression and role of physical or emotional violence on physical activity levels and body weight. Finally, just over half of ACCHS clients, particularly those aged over 55 yrs, may require assistance focusing mainly on diet and weight. Our results further suggest that social change focusing on employment and reducing stress and violence may produce additional health benefits for Aboriginal communities. Given these patterns, it should be possible to design and tailor programs which cater particularly for the client groups most likely to need them.

## Conclusion

Although multiple behaviour change interventions have shown potential for improving health, [16] evidence about their effectiveness remains limited, [18, 40] particularly for Aboriginal and Torres Strait Islander populations [47]. Further research including Aboriginal communities from a diversity of settings is required to establish whether the clustering patterns reported here are generalizable more broadly. If future research identifies similar stable clusters of health behaviours for this population, intervention approaches targeting these specific clusters of risk factors should be developed and tested for Indigenous Australians.

## Endnote

^1^Our sample included two participants of Torres Strait Islander origin and five participants of both Aboriginal and Torres Strait Islander origin. As the study was conducted in New South Wales, we have used the term ‘Aboriginal’ refer to all of the Aboriginal and Torres Strait Islander participants, following the guidelines of the New South Wales (NSW) Department of Health, in recognition that Aboriginal people are the original inhabitants of NSW [35].
